# Use of upcycled biosolids for bioremediation of groundwater contaminated with chlorinated solvents

**DOI:** 10.1007/s11356-025-37326-y

**Published:** 2026-01-08

**Authors:** Shahrzad Saffari Ghandehari, Isabelle Van Benschoten, Patricia D. Arcellana, James R. White, Cathleen Hapeman, Alba Torrents, Birthe Veno Kjellerup

**Affiliations:** 1 Department of Civil and Environmental Engineering, University of Maryland, College Park, MD USA; 2 Resphera Biosciences LLC, Baltimore, MD USA; 3 US Department of Agriculture, Agricultural Research Service, Beltsville, MD USA; 4 Present Address: Yokogawa Corporation of America, Sugar Land, TX USA

**Keywords:** Trichloroethene, Dehalogenation, Groundwater, Bioremediation, Biosolids, PICRUSt2, Organic waste, Wastewater

## Abstract

Trichloroethene (TCE) is a widespread toxic groundwater contaminant; thus, the development of inexpensive and accessible remediation materials is needed. Bioremediation of TCE under field conditions often requires the addition of carbon amendments. This study examines the use of biosolids at a field site as a sustainable carbon-rich and microbial amendment. A trench with amendments was installed upstream of a TCE-contaminated site, which was filled with biosolids, limestone, and biochar. Field monitoring of water quality parameters was compared with microbial community changes in soil samples collected 9 months after amendment. The results showed that the TCE concentration decreased from 91 µg/L to below the detection limit, with the presence of TCE biodegradation products indicating anaerobic reductive dehalogenation occurring in the trench. Analysis of the microbial community data also provided predictions about the metabolic activities and showed the presence of acetogenic activity. This activity is required for complete TCE dehalogenation and suggests the presence of different dehalogenase enzymes that can contribute to the degradation of chlorinated contaminants. Thus, the application of the waste product for sustainable TCE bioremediation in the field was successful in decreasing the contaminant levels by upcycling an organic waste product (biosolids).

**Supplementary Information:**

The online version contains supplementary material available at 10.1007/s11356-025-37326-y.

## Introduction

United Nations (UN) has identified 17 sustainable development goals that urgently need to be addressed by all countries (United Nations 2015). Access to clean water and sanitation is among the most highly prioritized goals (United Nations 2015). Almost 2.2 billion people around the world are struggling to access clean drinking water (World Health Organization 2022). Climate change together with anthropogenic contamination of surface waters makes groundwater an important drinking water source, and therefore, the need for access to clean groundwater is more crucial than ever before (World Health Organization 2022).

Trichloroethene (TCE) is an aliphatic organic compound, which is one of the most frequently found groundwater contaminants around the world (Hendrickson et al. 2002; Wu et al. 2022; Zhang et al. 2015). TCE can cause many health problems, such as liver and kidney damage, and cancer (Chiu et al. 2013; Wu et al. 2022). Under anaerobic conditions, TCE can be reductively dehalogenated by bacteria to 1,1-dichloroethene (1,1-DCE), *cis* 1,2-dichloroethene (*cis*-DEC), *trans* 1,2-dichloroethene (*trans*-DCE), and vinyl chloride (VC) (Jones et al. 2006; Pant and Pant 2010; Wu et al. 2022), and among these, VC is the most toxic degradation product. Because VC is often degraded slower than TCE and only a few dehalogenating microorganisms are capable of degrading VC (often termed “VC stall”), VC can accumulate during groundwater remediation processes (Murray and Richardson 1993; Vogel and McCarty 1985; Wiedemeier et al. 1996; Chan et al. 2021; Wu et al. 2022). Therefore, despite extensive research in this field, TCE bioremediation still faces challenges and needs innovative solutions.

Disposing of biosolids from wastewater treatment has, in some countries, become an environmental challenge due to issues with the emission of greenhouse gases, landfill disposal, methane production, and disposal costs (Dijkgraaf and Vollebergh 2003; Kulikowska & Klimiuk 2008; Rabl et al. 2008; Renou et al. 2008; Marchuk et al. 2023). However, biosolids can contain valuable organic matter, vitamins, microorganisms, and enzymes, which can be utilized if this product is upcycled (Singh and Agrawal 2008; Crocker et al. 2018; Paz-Ferreiro et al. 2018). Some of the current uses of biosolids include agricultural land applications, orchard fertilization, and remediation of contaminated soil. In the USA, specific requirements are present for each type of application (Chaney et al. 2000; Ginocchio et al. 2013; Paz-Ferreiro et al. 2014; Elgarahy et al. 2024). When upcycled and used for bioremediation of groundwater, this low-cost, or even free, beneficial waste material can be a source of nutrients, energy stored in chemical bonds, and microorganisms and can decrease the economic and environmental problems associated with landfilling and incineration (Surampalli et al. 2008; Sharma et al. 2017). This low-cost material and solution may also be accessible in regions and countries without access to high-cost bioremediation approaches, thus providing a sustainable solution and potentially access to cleaner groundwater.

The overall goal of this study was to assess the applicability of class A biosolids originating from municipal wastewater treatment as a sustainable bioremediation approach for a TCE-contaminated groundwater site, by biostimulation. In this field study, a trench containing biosolids, limestone, and biochar was installed to enhance biological TCE dehalogenation upstream of a previously installed biowall at a site in Beltsville, Maryland, USA, known as the Beaver Dam Road Land Fill (BDRLF). Microbial communities from the site were characterized, and the impact of the mixed trench filling material on the indigenous microbial community of groundwater, including the dechlorinating bacteria, was assessed (for more information on the indigenous microbial community of the site, please see Saffari Ghandehari et al. (2023, b). The metabolic and functional characteristics of the microbial communities were examined and compared with observed site activities. This study is among the first to investigate a field application of upcycled biosolids for bioremediation of chlorinated organic compounds in groundwater.

## Methods and materials

### Site description

BDRLF site is one of the hundreds of TCE contaminated sites in the USA (ATSDR 2019). It is located in Maryland, USA, and was used as a landfill from the 1940 s until the 1980 s (USDA ARS 2011). The site was added to the national priority list (NPL) for clean-up in 1994 and is under regular monitoring required under the Comprehensive Environmental Response, Compensation and Liability Act (CERCLA). The TCE plume is located downgradient from the landfill and is moving toward a small creek. In 2013, a permeable reactive barrier (PRB), also known as a biowall, was installed with compost, sand, and mulch, between the plume and the creek to promote the activity of dehalogenating bacteria and enhance TCE dehalogenation in the site (Niño de Guzmán et al. 2018a, b; Saffari Ghandehari et al. 2023a, b). The monitoring data from 2013 to 2019 showed that while there is a significant decrease in the TCE concentration, the VC concentration downgradient from the PRB had increased, indicating incomplete dechlorination of chlorinated solvents by the microbial community in the PRB.

The low groundwater residence time in the PRB (between 31 and 60 days for the TCE plume), together with low pH levels (an average of 6 for the PRB and 4.6 for the upgradient locations), was identified as the main limiting factor leading to the incomplete TCE dehalogenation. To increase the efficiency of the bioremediation process and to increase the residence time, a smaller trench was installed in 2020, approximately 9 m upgradient from the PRB, which is the focus of this study. The trench was filled with limestone, biosolids from wastewater treatment, and biochar (49%, 49%, and 2%, respectively) to increase the buffering and adsorption capacities of the trench and to increase the organic carbon and nutrients available to the microbial community. Biosolids were acquired from Blue Plains Advanced Wastewater Treatment Plant, Washington, D.C., and the toxicity characteristics leachate procedure (TCLP, SW-846 test method 1311, US EPA, 2015) was conducted, in a commercial laboratory, to ensure the safety of the waste product to be used based on the Resource Conservation and Recovery Act (RCRA) levels. Biosolids used in this study go through thermal hydrolysis and digesters to meet the most stringent requirements and are available commercially as Bloom soil amendment and are pathogen free (Barber 2017; Wang et al. 2018; Bloomsoil.com 2022). The material was mixed with limestone and tested in bench-scale studies prior to its installation (Saffari Ghandehari et al. 2023a, b) and was shown to enhance TCE dehalogenation.

### Collection of soil samples

**Fig. 1:**
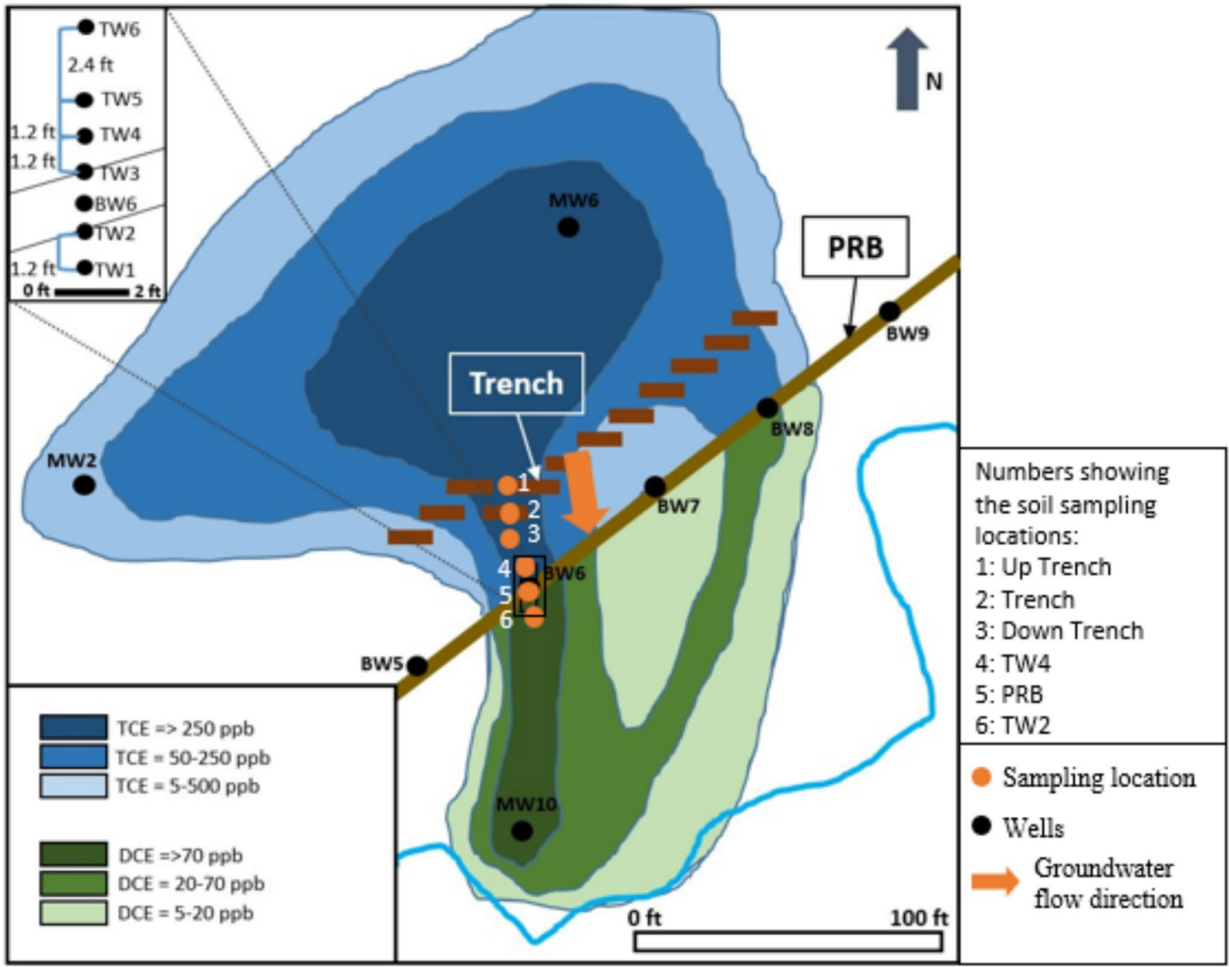
Map showing the BDRLF site, location of the PRB, trench, and the monitoring wells as well as the plume and the soil sampling locations are shown. TW, transect well; BW, biowall well (modified from BMT Designers & Planners Inc., 2018)

Soil samples were collected from six locations at the BDRLF site in August 2020 to characterize the microbial community of the soil and investigate the effect of the trench filling material on the indigenous microbial community. Soil samples were collected from the saturated zone: (1) upgradient of the trench (up trench), (2) the trench, (3) downgradient from the trench (down trench), in between the trench and the transect well 4 (TW4) upgradient from the PRB, (4) TW4, (5) in the PRB, and (6) transect well 2 (TW2) downgradient from the PRB (Fig. 1). All samples were collected using the GeoProbe (GeoProbe systems, Salina, KS) with the direct push method (Niño de Guzmán et al. 2018a, b). The soil samples from two different depths were recovered around the PRB area: (1) 2.0–2.4 m below the soil surface section and (2) 3.4–3.7 m below the soil surface section. The samples were cut into approximately 30 cm sections in the field, immediately wrapped in plastic bags to avoid evaporation, and transferred to the laboratory on ice. The samples were homogenized and stored in 50-mL centrifuge tubes at −80 °C prior to further processing.

Groundwater samples from the site were collected by a third party (USDA Collaborator) for analysis of volatile organic compounds (VOCs) levels, as well as data on the physical and geochemical characteristics of the groundwater. Samples for VOC measurements were collected in 40 mL vials with no headspace, preserved on ice, and analyzed in a commercial laboratory with USEPA method SW8260B (US EPA 1996). The results were reported to US EPA and USDA collaborator. UMD was also provided with the same format of data, presented in Fig. 3. Physical parameters including pH and temperature were measured by Horiba® U-52 Multiparameter Water Quality Meter (Horiba, Kyoto, Japan) (BMT, July 2017). The results were reported quarterly and were used in this study.

### Characterization of the microbial community

#### DNA extraction

0.25 g of soil was used for DNA extraction with DNeasy PowerSoil Kit (QIAGEN, Hilden, Germany), following the manufacturer’s protocol. To ensure the quality and concentration of extracted nucleic acids, the content was characterized by a NanoDrop 2000 UV–VIS spectrophotometer (Thermo Fisher Scientific, Waltham, MA). One gram of the soil sample (in triplicates) was weighed and dried at 106 °C for 24 h to measure the moisture content of the soil, which was used to normalize the concentration of nucleic acids based on dry soil weight.

#### Quantitative PCR

The abundance of dehalogenating bacteria was performed with Quantitative Polymerase Chain Reaction (qPCR) using primers targeting universal regions of 16S rRNA, and 16S rRNA of *Dehalobacter* spp. and *Dehalogenimonas* spp. (Table 1). West Branch Channel (WBC-2) culture (Jones et al. 2006), containing *Dehalobacter*, *Dehalogenimonas*, and *Dehalococcoides*, was used as a positive control for PCR and to prepare an eight-point standard curve using the serial dilution method for qPCR. All qPCR reactions were performed with a 25-µL total reaction volume and using 10.1µL of RT-PCR grade water (Ambion, Austin, TX, USA), 12.5 µL of SsoAdvanced Universal SyberGreen Supermix (Bio-Rad Laboratories, Hercules, CA, USA), for a final concentration of 0.4 µM of each of the forward and reverse primers, and 2 µL of template DNA. Negative controls were set up in each run with RT-PCR grade water (Ambion, Austin, TX, USA) and extracted DNA from *Escherichia coli* (*E. coli*). For each qPCR run, the melt curves were evaluated to confirm the presence of one peak and eliminate the possibility of nonspecific binding. The standards were loaded to the qPCR plate in triplicate; all values fell within the standard curve range for each qPCR run (Table 1).

**Table 1:** PCR thermal cycler protocols, primers used, and calibration curve range

**Targeting gene**	Primer	Sequence	Thermal cycler program	Reference	Standard curve range (gene copies/µL)
16S rRNA Universal	341 F	5′-CCTACGGGAGGCAGCAG-3′	5 min at 94 °C, 60 s at 94 °C, 60 s at 60.2 °C, 10 s at 72 °C, 40 cycles	(Gurtner et al. 2001; Ishii and Fukui 2001)	1.76 × 10^3^–1.76 × 10^10^
	907R	5′-CCGTCAATTCCTTTRAGTTT-3′			
16S *Dehalobacter* spp*.*	DHb 477 F	5′-GATTGACGGTACCTAACGAGG-3′	95 °C for 3 min, 40 cycles of 95 °C for 15 s, 60 °C for 30 s	(B. J. K. Smith et al., 2015)	3.61 × 10–3.61 × 10^9^
	Dhb 647 R	5′-TACAGTTTCCAATGCTTTACG-3′			
16S *Dehalogenimonas* spp*.*	Dhg 634 F	5′-GGTC ATCTG ATACTGTTGG ACTTG AGT ATG-3′	95 °C for 3 min, 40 cycles of 95 °C for 15 s, 60 °C for 30 s	(B. J. K. Smith et al., 2015)	2.31 × 10^3^–2.31 × 10^11^
	Dhg 799 R	5′-ACCCAGTGTTT AGGGCGTGG ACT ACCAGG-3′			

#### Illumina MiSeq sequencing

15 µL of the extracted DNA was sent to the Institute of Marine and Environmental Technology (IMET) for Illumina MiSeq sequencing (BioAnalytical Services Laboratory (BASLab) 2025). The concentration of nucleic acids ranged from 2.4 to 5.7 ng/µL, and the samples with higher concentrations were diluted to fit in this range. V3–V4 hypervariable regions of 16S rRNA were targeted during the sequencing process (Cai et al. 2013).

The DADA2 plug-in (Callahan et al. 2016) in Quantitative Insights Into Microbial Ecology 2 (QIIME 2, V.2020.11) (Bolyen et al. 2019) was applied to filter and denoise the acquired data, with 10 nucleotides being removed with trim-left and truncation at position 120, upon evaluating the quality plots. A customized database (Saffari Ghandehari et al. 2023a, b) was applied that was prepared by removing the low-quality sequences and taxa associated with uncultured and unidentified from the SILVA v138 database (Quast et al. 2013; Yilmaz et al. 2014) for a taxonomy assignment with 97% similarity. A sampling depth of 16,000 reads was used for rarefaction to reach an even sampling depth for diversity analyses, based on the developers’ suggestions and to be able to include all the samples in the analysis. RStudio (V 4.0.3) was used to prepare unweighted UniFrac distance matrices and principal coordinate analysis (PcoA) plots based on the calculated distance matrix (Lozupone and Knight 2005).

### Prediction of activity using the phylogenetic data

**Fig. 2:**
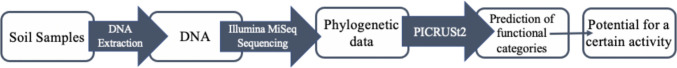
Processing of the soil samples and application of bioinformatics tools in this study. PICRUSt2 can be used to predict the potential for a metabolic pathway in the soil samples

An investigation of the communities by reconstruction of unobserved states (PICRUSt2, Douglas et al. 2020) was used in QIIME2 to predict the metabolic pathways in the microbial community using the 16S rRNA sequencing results (Fig. 2). Relative abundances of enzyme commission numbers (EC numbers) and Kyoto Encyclopedia of Genes and Genomes (KEGG) orthologs (KO numbers) for selected targets including dehalogenation, acetogenesis, and methanogenesis were calculated. The relative abundance of each taxon or EC number was used to prepare heatmaps in RStudio. To predict the dehalogenation potential in the soil samples, the PICRUSt2 database was filtered for dehalogenase enzymes and associated KO and EC numbers to include microorganisms that are capable of dehalogenation (Copley 1998; Ang et al. 2018). Among the reductive dehalogenation enzymatic classifications in the KEGG database (PceA and PceB, EC: 1.21.99.5) responsible for tetrachloroethene reduction, none was listed in the PICRUSt2 database. However, some of the hydrolytic dehalogenase enzymes were listed in the database and were used in this study (Ang et al. 2018). The heatmap with the selected KO numbers is shown in Fig. S2 in the supplementary information.

### Statistical analysis

RStudio (V 4.0.3) was used to conduct the statistical analysis in this study. One-way ANOVA tests were performed to assess if differences between bacterial abundances (qPCR results) in different locations in the site were statistically different (with a confidence interval of 95%). A *t*-test was conducted between the trench and up trench samples to determine a significant difference between each other in terms of gene copy numbers of the dehalogenating bacteria (with a confidence interval of 95%). Permutational multivariate analysis of variance (PERMANOVA) test was also performed to analyze the differences between the samples for the sequencing results, using an unweighted UniFrac distance matrix and via vegan package (Oksanen et al. 2020).

## Results and discussion

### Analysis of the trench and surrounding area

**Fig. 3:**
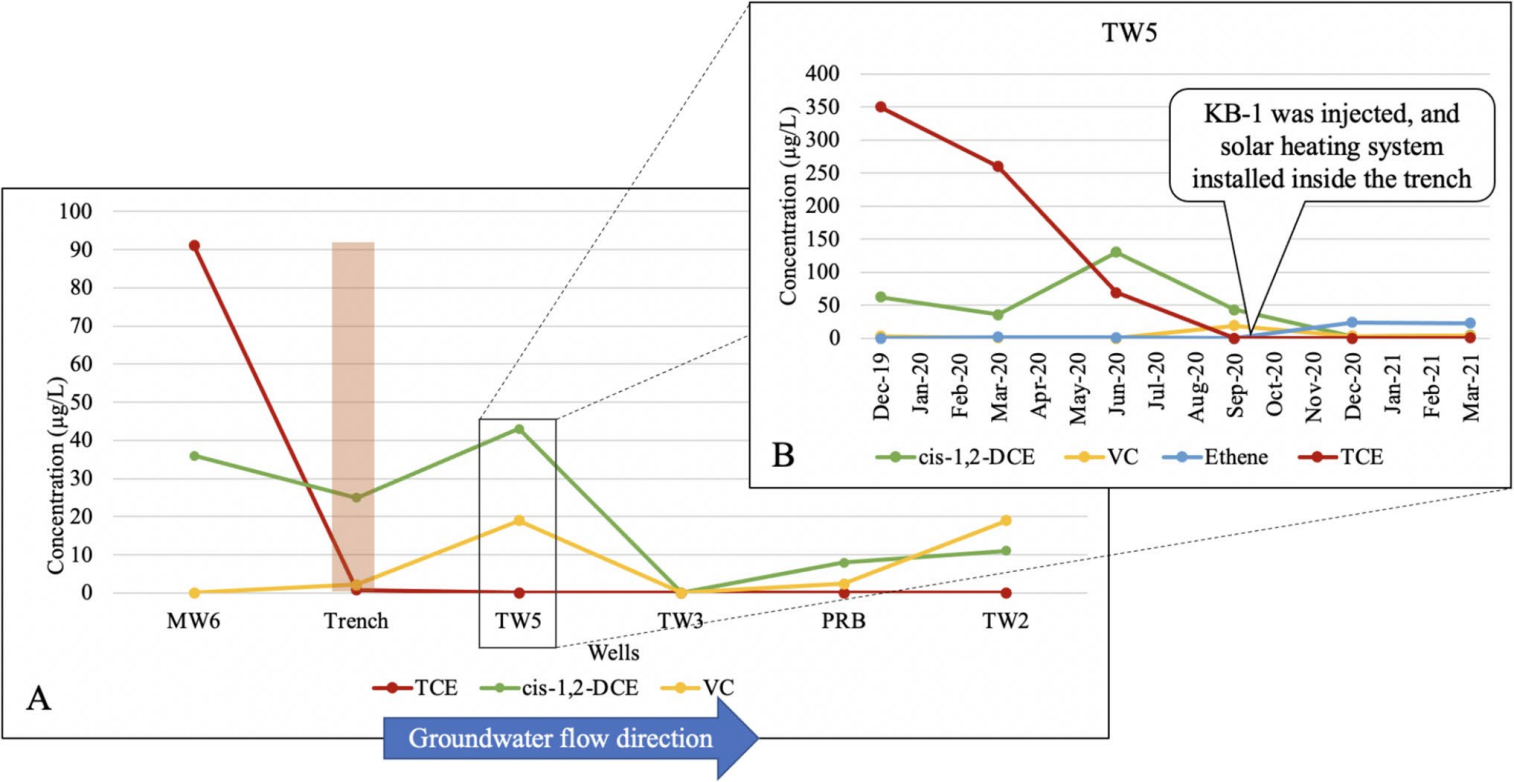
**A** Concentration of chlorinated solvents, measured in groundwater samples, inside wells, from upgradient the trench to downgradient the PRB, 9 months after installation of the trench (September 2020 monitoring event). **B** Concentration of the chlorinated solvents over time, in one well (TW5) between the trench and the PRB (in groundwater samples)

Soil samples were collected before the trench was installed, and monitoring data were collected for 9 months after the installation. Results showed that the TCE levels had decreased notably when groundwater encountered the trench (from 91 µg/L upgradient to below detection limits inside the trench), and concentrations remained low down trench (Fig. 3A). Monitoring results from installation of the trench to 9 months later showed a decrease in the TCE concentration down trench and an increase in TCE degradation products, which strongly suggests that reductive dehalogenation of TCE is occurring in the trench.

The feasibility of using biosolids as an amendment for groundwater bioremediation has been previously documented (Saffari Ghandehari et al. 2023a, b). The benefit of using the biosolids amendment is that it supplies organic matter, nutrients, enzymes, cofactors, and potentially a dehalogenating microbial community that all can improve the ability of TCE dehalogenation in the indigenous soil microbial community. Other studies such as Lu et al. (2021) have also shown that the use of waste activated sludge can enhance the dehalogenation in tetrachloroethene (PCE)-contaminated soil by both bioaugmentation and biostimulation of the microbial community (Q. Lu et al. 2021). They also suggested that volatile fatty acids and consequent H_2_ release from the amendment could act as electron donors while providing the dehalogenating bacteria with essential cofactors for metabolism (Q. Lu et al. 2021).

TW5 is the transect well located approximately 8 m downgradient from the trench and is between the trench and the PRB. The groundwater velocity in the vicinity of transect wells was originally estimated to be around 9–15 m/year and, accounting for the retardation factor in the site (1.2–1.5), the TCE migration velocity was predicted to be 5.5–10.7 m/year (BMT Designers and Planners Inc., 2017). Therefore, 9 months after the installation of the trench, the groundwater would have theoretically travelled 6.8–11.3 m, and even the TCE plume in the trench groundwater would have traveled around 4.3 to 8 m downgradient and toward the PRB, and most likely it had affected TW5. Monitoring results for TW5 showed a decrease in the TCE concentration of the groundwater flowing through this well, as well as an increase in the concentration of TCE dehalogenation products by 9 months, which indicated that the dehalogenation process was happening at that time (Fig. 3B).

The trench was bioaugmented after 9 months with KB-1 (an anaerobic dehalogenating mixed culture, SiREM Lab, Guelph, Ontario), and a solar heating system was installed inside the trench. KB-1 is a consortium of bacteria that has been enriched from the soil samples of a TCE-contaminated site in Ontario, Canada. *Dehalococcoides mccartyi*, *Geobacter*, and *Dehalobacter *are the major dechlorinating bacteria that are present in this consortium (Molenda 2018). KB-1 also includes fermenting, acetogenic, methanogenic, and dechlorinating organisms (Molenda 2018). At 12 months, the concentrations of DCE and VC decreased to below detection and 2.7 µg/L, respectively (from initial levels of 43 and 19 µg/L, respectively), while ethene levels rose to 23 µg/L (from the initial below detection limit value at 9 months). Based on the calculated groundwater velocity (highest groundwater velocity of 15 m/year), it is very unlikely that the groundwater affected by these two additional treatments at 9 months reached TW5 at 12 months. Thus, the decrease in DCE and VC concentrations and the increase in ethene levels is most likely the effect of trench filling material. These observations indicate that the trench and the filling material were successful in decreasing the TCE concentration, which would subsequently reach the PRB further downgradient from the trench and show that the trench filling material has enhanced overall TCE dehalogenation in the site.

### Impact of trench filling material on soil microbial community

**Fig. 4:**
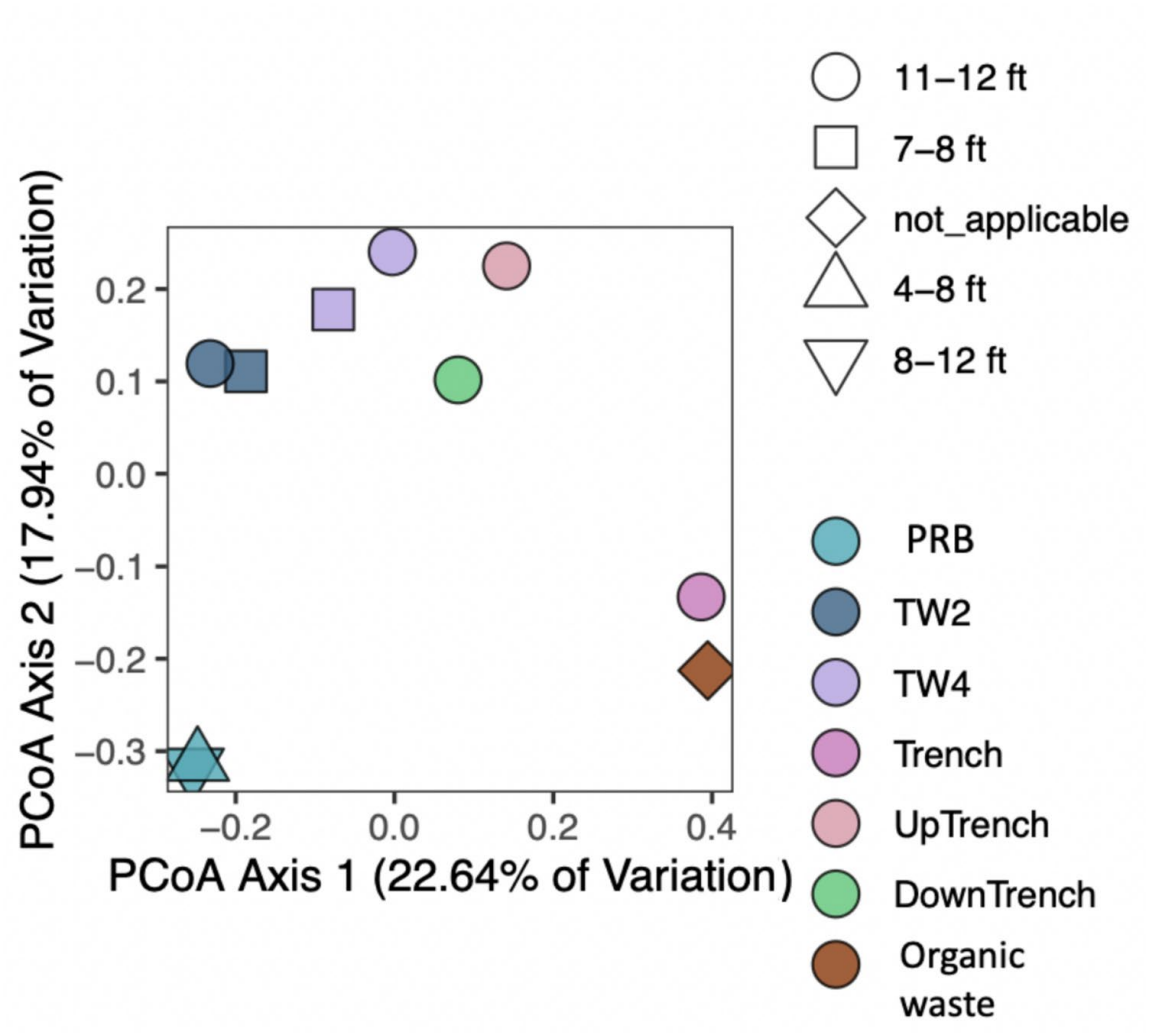
The similarity of microbial communities in soil samples from the site. Determined by PCoA analysis based on unweighted UniFrac dissimilarity matrix. Different color shades are referring to each sampling location, whereas the different shapes are referring to the depths at which the sample was taken

The microbial community of the trench filling material seems to be most similar to biosolids and thus indicating the contribution of the amendment to the trench microbial community. There was a significant difference between microbial communities of the soil collected from different locations in the site (*p* < 0.05); however, the depth of the samples did not appear to affect the microbial community significantly (*p* > 0.05, Fig. 4). This can be explained based on the collapse of the soil inside the cores and not being able to collect distinctive layers of the soil from different depths. It could also be due to the similarity of physical and geochemical conditions in these layers, which would have produced a similar microbial community throughout the soil profile.

Among the top 100 most abundant taxa (Fig. S1, supplementary information), eight were dehalogenating bacteria, seven were acetogens, and seven are methanogens. Among these taxa, *Methanosaeta*, *Methanohalophilus*, *Methanobacterium*, *Methanospirillum*, *Methanosarcina*, *Methanoregula*, and *Methanoculleus* are methanogens. *Acetobacterium*, *Sporomusa*, and *Eubacterium* are considered to be acetogens. Similarly, *Dehalococcoides*, *Dehalogenimonas*, and *Dehalobacter* are the dehalogenating bacteria found in the samples. Acetogens and methanogens are naturally present in the environment and can serve an important role in a balanced dehalogenating ecosystem by providing co-factors, enzymes, electron donors, and carbon sources necessary for dehalogenation. Acetogens are able to produce acetate, which can be utilized as an organic compound by dehalogenating bacteria such as *Dehalococcoides* (Puentes Jácome et al. 2019). In addition, the vinyl chloride reductase (VcrA) is only active when cobalamin (vitamin B_12_) is present (Puentes Jácome et al. 2019). And vitamin B_12_ is produced when 5,6-dimethylbenzimidazole (DMB) is the lower axial ligand of cobamide, which some acetogenic bacteria can produce, such as members of the *Eubacteriaceae* family including *Eubacterium limosum* and *Acetobacterium woodii* (Puentes Jácome et al. 2019). Thus, a larger relative abundance of the genera *Eubacterium* and *Acetobacterium* in biosolids samples may indicate support for the dehalogenation process by these microorganisms.

**Fig. 5:**
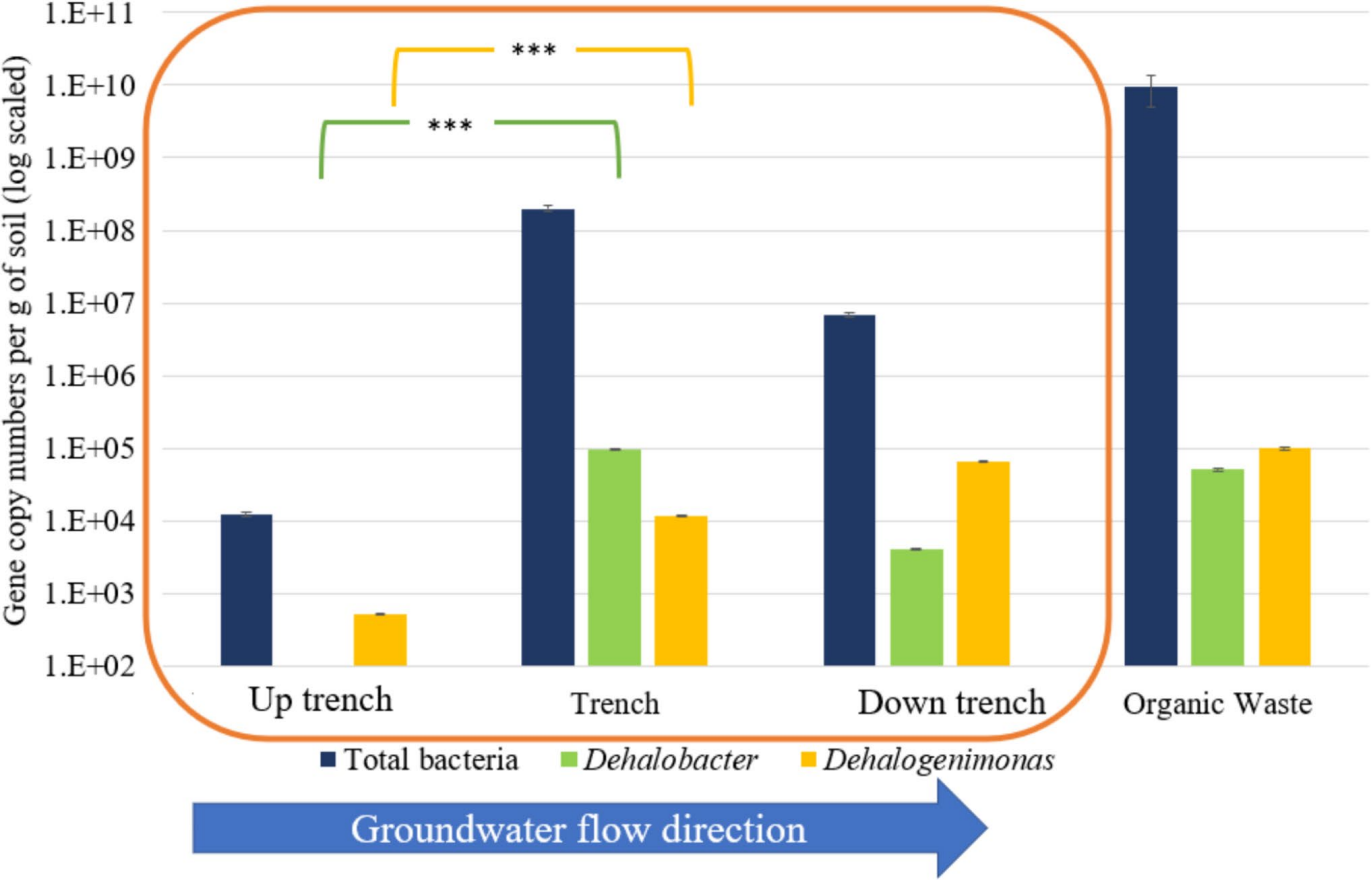
*Dehalobacter* spp. 16S rRNA gene copy numbers and *Dehalogenimonas* spp. 16S rRNA gene copy numbers found in each location in the site, compared to the total number of bacteria. The error bars indicate the standard error between the triplicates in each qPCR well. Significance level: *** < 0.001

The abundance of *Dehalobacter* spp. at 9 months was significantly higher in the trench samples compared to samples collected up trench at the site (Fig. 5). This is not surprising since biosolids contained a higher abundance of *Dehalobacter* spp. per g of sample, compared to the up trench soil. The soil samples from the trench and down trench also had a significantly higher abundance of *Dehalogenimonas* compared to the up trench soil samples (Fig. 5). Biosolids had a high abundance of *Dehalogenimonas* compared to other soil samples, thus the organic amendment had a positive contribution to the abundance of *Dehalogenimonas* in the trench and down trench.

The absolute abundances of bacteria in biosolids and trench samples (9 × 10^9^ and 2 × 10^8^ gene copies of 16S rRNA, respectively) were significantly higher than in the up trench sample (10^4^ gene copies of 16S rRNA), which might explain the lower relative abundance of *Dehalogenimonas* and *Dehalobacter* in the trench samples (Fig. 5). It is likely that the presence of chlorinated compounds in wastewater (Jing and Kjellerup, 2020) could have caused a high abundance of *Dehalobacter* and *Dehalogenimonas* in the biosolids and consequently contributed to the higher abundance of dehalogenating bacteria inside the trench.

### Prediction of metabolic pathways

**Fig. 6:**
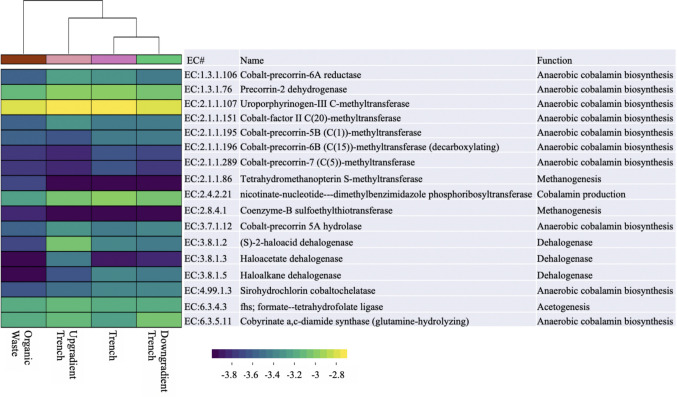
Heatmap showing the relative abundance of relevant classification of enzymes as predicted by PICRUSt2. The scale shows the logarithm of the relative abundance value. The hierarchical clustering on the top shows how the samples cluster together based on the Euclidean similarity

To understand the potential metabolic pathways of the microbial community better in the soil and biosolids samples, PICRUSt2 was used to predict the functional characteristics based on the phylogenetic sequencing results. Prediction of metabolic and functional categories was performed to assess the abundance of acetogenic activity in the samples since acetogenesis is a physiological function rather than a phylogenetic trait (Müller and Frerichs 2013). The gene *fhs* encodes for formate-tetrahydrofolate ligase and has been used for detection of acetogenic bacteria (Müller and Frerichs 2013). The analysis showed that the predictive relative abundance of *fhs* did not change for the samples (Fig. 6), which indicates that acetogenic bacteria were present in all samples.

#### Anaerobic pathway of cobalamin production to vitamin B_12_

As mentioned in Sect. “Impact of trench filling material on soil microbial community”, Vitamin B12 is essential for vcrA activation and thus plays an important role in the complete degradation of TCE (Puentes Jácome et al. 2019). The anaerobic pathway of cobalamin production is a complex pathway with around 30 different genes involved (Balabanova et al. 2021; Hazra et al. 2015). Not all the pathways are well understood, but some of the common pathways were tracked here (Hazra et al. 2015; Balabanova et al. 2021). The enzymatic categories that are involved in the anaerobic production of the cobalamin (Hazra et al. 2015; Balabanova et al. 2021) are shown in Fig. 6.

Up trench and down trench samples have a lower relative abundance of EC: 2.1.1.196 and EC: 2.1.1.289, compared to the trench and biosolids sample, which may be due to the effect of the biosolids on the microbial community of the trench. The gene *cobT* (EC: 2.4.2.21) is also involved in the cobalamin production pathways (Hazra et al. 2015; Balabanova et al. 2021), and it was predicted to be present in all samples. Overall, all the samples had the potential for the production of cobalamin with DMB as a lower ligand, which is necessary for the vinyl chloride reductase enzyme to catalyze the dehalogenation process (Puentes Jácome et al. 2019; Wen et al. 2020). Thus, the potential for complete dehalogenation is present at this site, and the VC stall may be less likely.

#### Predicting dehalogenation pathways

Six dehalogenation mechanisms are known: (1) reductive dehalogenation, (2) hydrolytic dehalogenation, (3) haloacid dehalogenation, (4) haloalkane dehalogenation, (5) fluoroacetate dehalogenase, and (6) 4-chlorobenzoyl CoA dehalogenase (Ang et al. 2018). KEGG database and PICRUSt2 were used to predict the relative abundance of different dehalogenation pathways here. Among the eight KO numbers, which were associated with dehalogenation in the PICRUSt2 database, 2-haloacid dehalogenase (EC:3.8.1.2, K01560), haloacetate dehalogenase (EC: 3.8.1.3, K01561), and haloalkane dehalogenase (EC:3.8.1.5, K01563) were detected in the samples.

These enzymes all belong to the hydrolytic dehalogenation group, where a water molecule is necessary to break down halogenated compounds. They are categorized in a different branch of dehalogenase enzymes than reductive dehalogenase (*rdhA*), which are mostly responsible for reductive dehalogenation of chlorinated ethenes (Ang et al. 2018). Unfortunately, none of the RdhA enzymes was represented in the PICRUSt2 database. Therefore, the information that we obtained from PICRUSt2 regarding anaerobic reductive dehalogenation was very limited.

2-haloacid dehalogenase and haloalkane dehalogenase were predicted to have higher relative abundance in all of the samples, compared to the haloacetate dehalogenase. The haloalkane dehalogenase, haloacid dehalogenase, and 2-haloacid dehalogenase are non-respiratory dehalogenation enzymes, mostly belonging to bacteria that are not obligate dehalogenating bacteria (Temme et al. 2019). Again, prediction of the presence of dehalogenating enzymes capable of degrading different halogenated compounds (Ang et al. 2018) in the biosolids samples is likely due to the presence of halogenated compounds in the wastewater (Jing and Kjellerup, 2020). We surmise that the microbial community adapts to the available substrates and potentially enhances the dehalogenation activity of the microbial community within the soil. However, at this stage, it is hard to prove or disprove the direct impact of these organisms on TCE degradation within the site.

#### Role of methanogens

Methanogens can use a variety of metabolites to produce methane, but one of the most common pathways is using hydrogen and carbon dioxide (Thauer et al. 2008). Considering that hydrogen is also the sole electron donor used by dehalogenating bacteria, methanogens and dehalogenating bacteria can potentially compete (Henry 2008; Mueller and Booth 2016). However, methanogens are also capable of producing enzymes and cofactors necessary for the dehalogenation process (Fathepure and Boyd 1988; Jones et al. 2006; Men et al. 2013; Wen et al. 2015, 2020).

For methanogenesis, Coenzyme-B sulfoethylthiotransferase (MCR: methyl-coenzyme M reductase: EC: 2.8.4.1 and KO: K00399-K00402) and tetrahydromethanopterin S-methyltransferase (MTR: EC: 2.1.1.86, KO numbers K00577-K00584) are essential in methane production (Gottschalk and Thauer 2001; Luton et al. 2002; Jones et al. 2006; Steinberg and Regan 2008; Munk et al. 2010). Among the samples collected from the trench area, the predicted relative abundance of these enzyme complexes was very low compared to other enzymatic categories investigated in this study. Relative abundances of EC: 2.8.4.1 and EC: 2.1.1.86 are 2 to 3 orders of magnitude less than the average of all the EC numbers mentioned here, both inside the trench and down the trench. These results show that the methanogenic activity was not predicted to be dominant in the trench with the biosolids amendment. Thus, competition was not likely to have an impact on dehalogenation. It should be noted that these predictions are based on the relative abundance of different taxa in the soil samples. Furthermore, it is likely that the absolute abundance of the predicted enzymatic categories is different based on the absolute abundance of the bacterial cells present in the samples (Fig. 5).

Overall, the PICRUSt2 database is limited for the purpose of analyzing metabolic pathways of thedehalogenating bacteria; however, this analysis for methanogenesis, cobalamin production, and acetogenesis added valued information on the overall microbial analysis. Additional metatranscriptomics analysis can be used to overcome the limitations here; however, PICRUSt2 provides the opportunity of using data that was collected with the Illumina sequencing results, which is a relatively cheap and easy technique, to a greater extent. Further investigations are necessary, and the addition of dehalogenating enzymes to the PICRUSt2 database is also beneficial.

Overall, the microbial community of the trench was significantly altered by the biosolids amendment. Increased numbers of obligate dehalogenating bacteria and the increase in TCE dehalogenation down trench suggest a positive impact from the biosolids amendment on TCE dehalogenation at this site. As for the other trench filling materials, limestone was added to enhance the buffer capacity of the soil, while biochar was added to increase the sorptive capacity. Considering the effect of precipitation events and temperature changes, the TCE concentration in TW5 fluctuated between 1000 and 400 µg/L, from November 2013 to September 2019, before the installation of the trench, while it decreased to below detection values after its installation, which shows that the significant decrease in TCE concentration is directly related to the trench filling material. Although sorption to the biochar and the organic matter amendment might have contributed to reduced levels of TCE down trench, the presence of TCE dehalogenation products confirmed that dehalogenation was occurring in the trench.

Some concerns have been raised about using biosolids originating from wastewater treatment plants for land applications (Clarke and Smith 2011; Brooks et al. 2015). It has been shown that the biosolids from wastewater treatment plants can contain different organic chemicals that are a part of modern cities (Clarke and Smith 2011). Some of these chemicals are going to remain adsorbed to the biosolids and will not be mobilized in groundwater due to their high *K*_ow_ values (Schwarzenbach et al. 2003; Almeida e Andrade 2012). Inside the trench, these chemicals are likely to stay in the biosolids or adsorbed to biochar that is used in the trench. In addition, some of these contaminants can go through degradation, either abiotically or biologically (Lozano et al. 2010; Clarke and Smith 2011; Almeida e Andrade 2012). For example, wastewater can contain triclosan, a commonly used antibacterial agent found in pharmaceuticals and personal care products that can persist in soils (Fischer 2019). However, several studies have shown little impact on crop uptake in agricultural applications (Lapen et al. 2008; Gottschall et al. 2012; Mohapatra et al. 2016; Fu et al. 2016). These compounds are also biodegradable, and triclosan has shown to dissipate after a single land application, with a half-life of 107 days (Lozano et al. 2010). The presence of antibiotic resistance genes and bacteria in wastewater and thus the organic amendment is another potential topic of concern (D’Costa et al. 2006; Brooks et al. 2007). It has been shown that land application of the organic amendment did not increase the number of antibiotic-resistant bacteria in the soil above the natural background level (Brooks et al. 2007). The biosolids that were used in this study were cleared by US EPA to be used for agriculture and have gone through extensive treatments at high temperatures and pressures (*High-Quality Biosolids—Cambi* 2022). Leaching of organic matter from the amendment into the groundwater and consequently to surface water nearby should also be closely monitored to ensure that the bacteria in the site are utilizing the organic matter instead of the leaching contributing to eutrophication in the receiving waters.

In this study, the effect of biosolids amendment on the microbial community of a TCE-contaminated groundwater site as well as the overall dehalogenation process in the groundwater was investigated. The microbial community of the soil samples collected 9 months after installation of the trench was affected by the organic amendment. *Dehalobacter* and *Dehalogenimonas* spp. abundances were higher within the trench and/or down trench, compared to the up trench soil, which showed the positive effect of the organic amendment on the microbial community of the soil. Metabolic pathways were predicted within the soil samples to paint a clearer picture of the activity of the microbial community. Enzymes needed for cobalamin production were predicted to be present in all the samples, which supports the ability of the indigenous microbial community of the site in degrading TCE. In addition, excessive methanogenesis is not predicted in the trench sample, which could enhance the likelihood of complete TCE degradation.

The TCE concentration was significantly decreased down trench; thus, the organic amendment, together with lime and biochar, was effective in TCE dehalogenation and lowering the TCE levels down trench. The full-scale study documented the benefit of utilizing biosolids as a cheap, organic carbon and nutrient source in the bioremediation of chlorinated solvents in groundwater.

## Supplementary Information

Below is the link to the electronic supplementary material.

ESM 1 Supplementary Material 1 (DOCX 202 KB)

ESM 2 Supplementary Material 1 (DOCX 1.87 MB)

## Data Availability

Data will be shared upon request – please contact the corresponding author: bvk@umd.edu.
