# Supplementary material for: Use of upcycled biosolids for bioremediation of groundwater contaminated with chlorinated solvents
Source: Environ Sci Pollut Res Int. 2026 Jan 8;33(2):526–39. doi: 10.1007/s11356-025-37326-y (PMC12882858; doi:10.1007/s11356-025-37326-y)
Supplement: Supplementary file 2 — Supplementary Material 1 (DOCX 1.87 MB) [file 11356_2025_37326_MOESM2_ESM.docx]

**SUPPLEMENTARY INFORMATION**


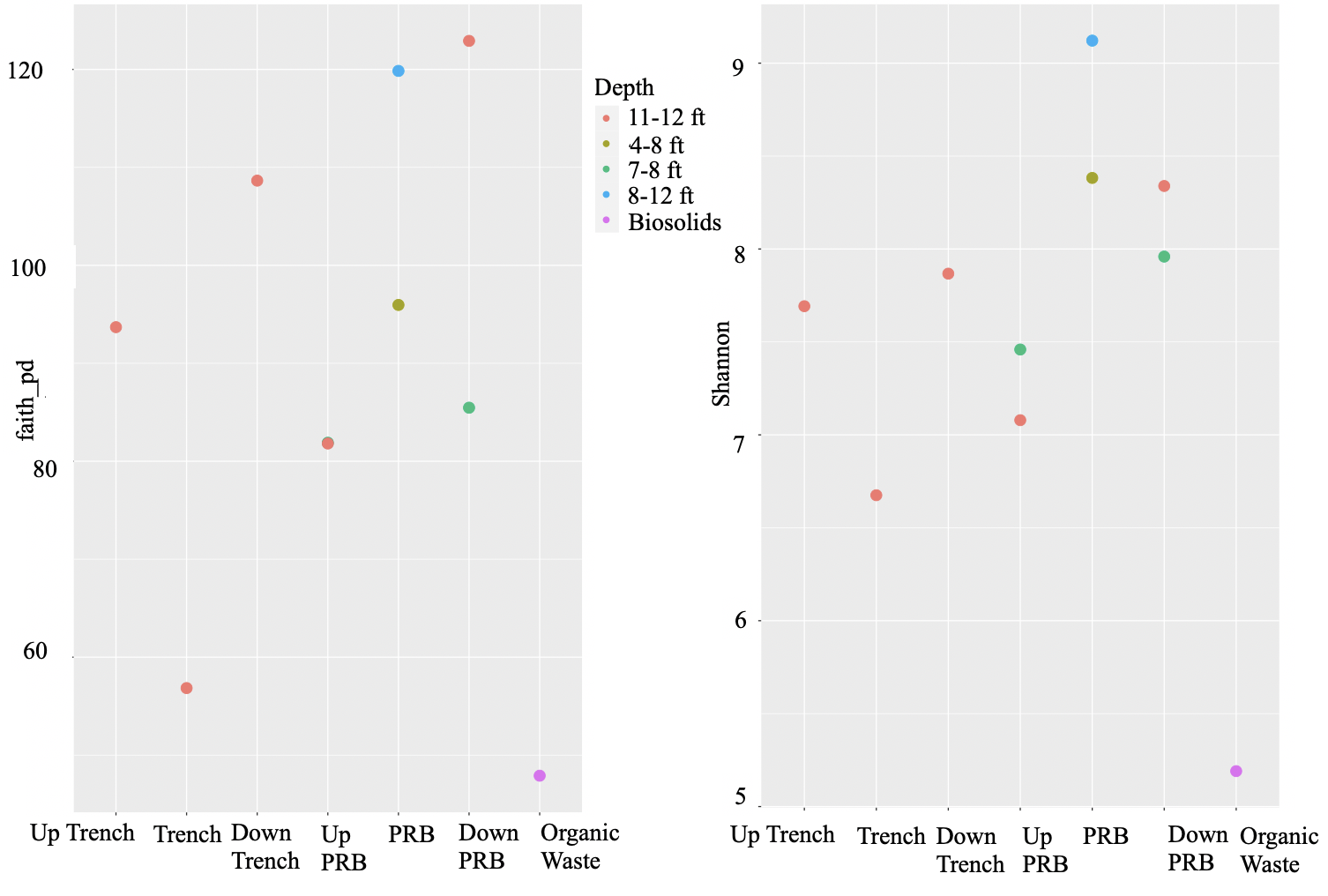


Figure S1. Alpha diversity in the samples determined by the Shannon Index and Faith Phylogenetic Diversity.


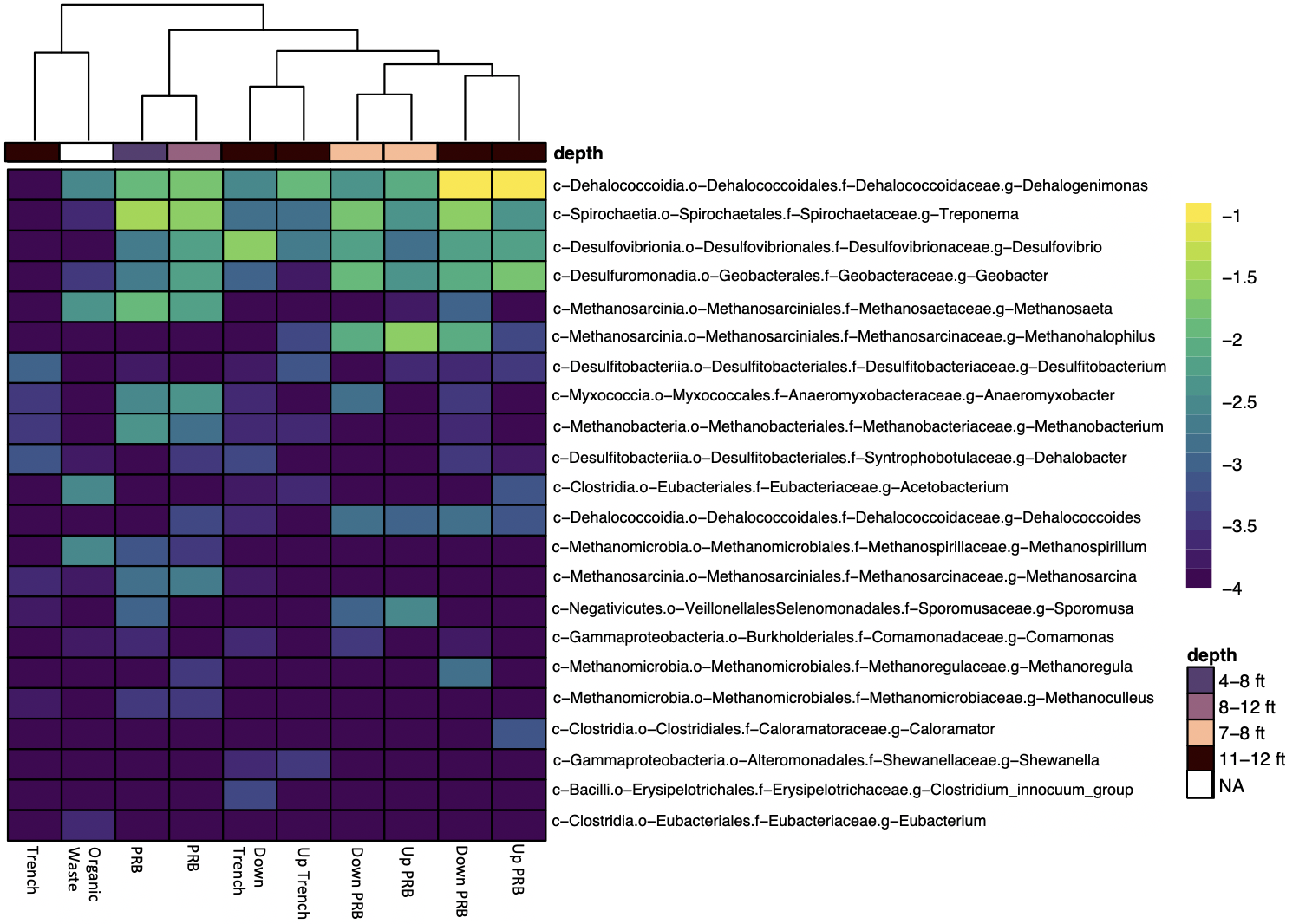


Figure S2. Heatmap showing the relative abundance of dehalogenating bacteria, acetogens, and methanogens among the top 100 most abundant taxa in our samples. The scale shows the logarithm of the relative abundance value. The hierarchal clustering on the top shows how the samples cluster together based on the Euclidian similarity.


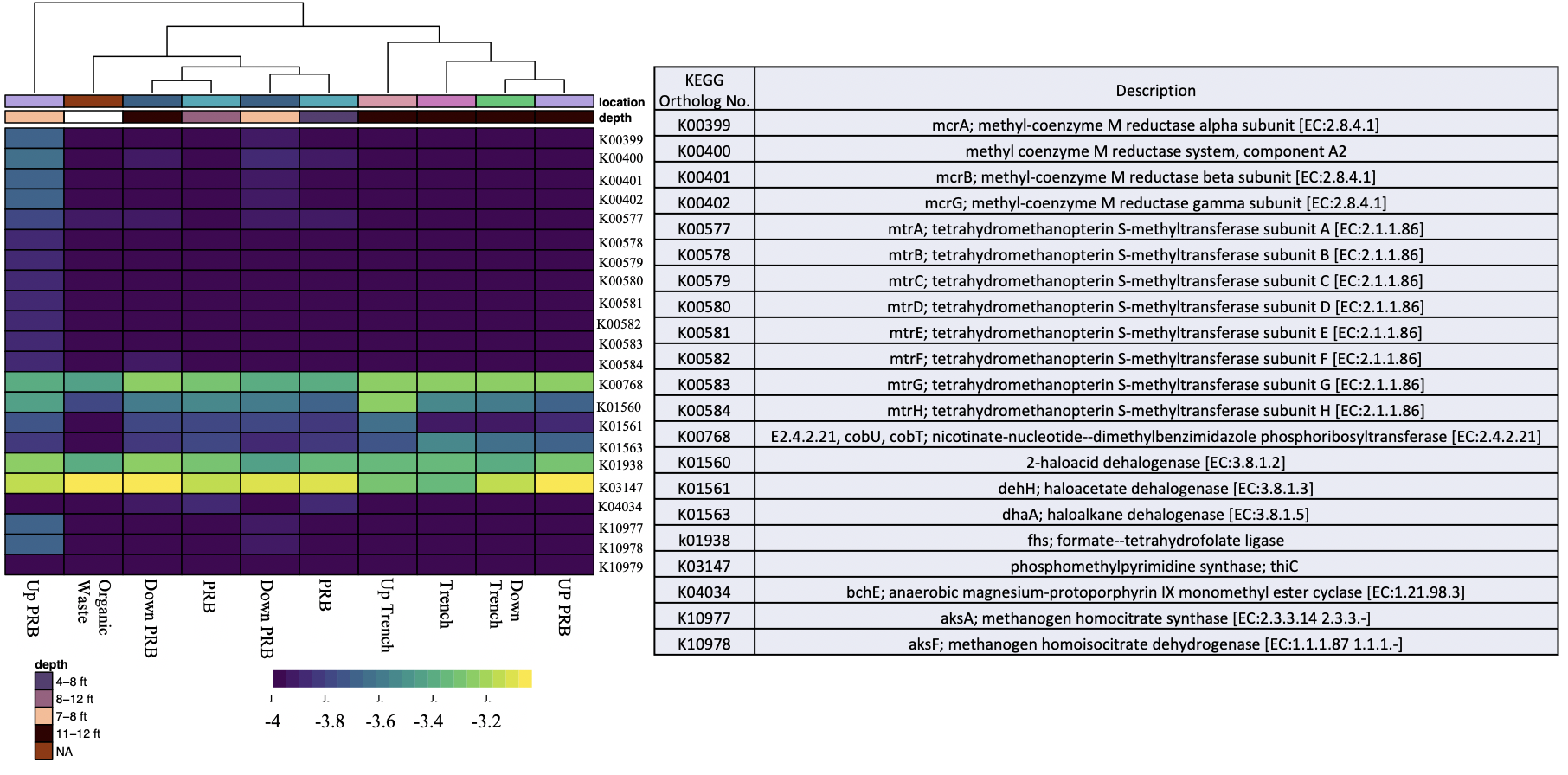


Figure S3. Heatmap showing the relative abundance of relevant EC numbers as predicted by PICRUSt2. The scale shows the logarithm of the relative abundance value. The hierarchal clustering on the top shows how the samples cluster together based on the Euclidian similarity.
