# Supplementary material for: Use of upcycled biosolids for bioremediation of groundwater contaminated with chlorinated solvents
Source: Environ Sci Pollut Res Int. 2026 Jan 8;33(2):526–39. doi: 10.1007/s11356-025-37326-y (PMC12882858; doi:10.1007/s11356-025-37326-y)
Supplement: Supplementary file 1 — Supplementary Material 1 (DOCX 202 KB) [file 11356_2025_37326_MOESM1_ESM.docx]

**Rebuttal - ESPR-D-24-13866- Reject with possible Resubmission**

*Environmental Science and Pollution Research*

Reviewers' comments:

**The responses to the comments are marked with [].**

Reviewer #1:

Although the manuscript could be of interest to the audience, several issues were present. - Presence of conflicting and confusing sentences (few examples provided).

-	Throughout the manuscript, please update references to within the last 6 years (if possible).

-	No details pertaining to how many replicates were used for each sample location.

[Thank you for this comment. We have revised the manuscript including the sentences that were brought to our attention. The changes are marked with Track-changes.]

Line 24-25 only need to define TCE once in the abstract

[Thank you for this comment. This has been addressed in the text.]

Line 48 please update references

[Thank you for your comment, a 2022 paper was added:

Wu, Z., Man, Q., Niu, H., Lyu, H., Song, H., Li, R., Ren, G., Zhu, F., Peng, C., Li, B., & Ma, X. (2022). Recent advances and trends of trichloroethylene biodegradation: A critical review. *Frontiers in Microbiology*, *13*. https://doi.org/10.3389/fmicb.2022.1053169]

Line 50 please update references

[Thank you for your comment, a 2022 paper was added:

Wu, Z., Man, Q., Niu, H., Lyu, H., Song, H., Li, R., Ren, G., Zhu, F., Peng, C., Li, B., & Ma, X. (2022). Recent advances and trends of trichloroethylene biodegradation: A critical review. *Frontiers in Microbiology*, *13*. https://doi.org/10.3389/fmicb.2022.1053169]

Line 52 please update references

[Thank you for your comment, a 2022 paper was added:

Wu, Z., Man, Q., Niu, H., Lyu, H., Song, H., Li, R., Ren, G., Zhu, F., Peng, C., Li, B., & Ma, X. (2022). Recent advances and trends of trichloroethylene biodegradation: A critical review. *Frontiers in Microbiology*, *13*. https://doi.org/10.3389/fmicb.2022.1053169]

Line 56 please update references

[Thank you for your comment, a 2022 and a 2021 paper were added:

Chan, A.-L., Tseng, C.-Y., Chen, T.-W., Lo, Y.-H., & Chang, S.-C. (2021). Microbiome Reengineering by Heat Selection for Rapid Biodegradation of Trichloroethylene with Minimal Vinyl Chloride Formation. *Water, Air, & Soil Pollution*, *232*(3), 121. https://doi.org/10.1007/s11270-021-05028-z

Wu, Z., Man, Q., Niu, H., Lyu, H., Song, H., Li, R., Ren, G., Zhu, F., Peng, C., Li, B., & Ma, X. (2022). Recent advances and trends of trichloroethylene biodegradation: A critical review. *Frontiers in Microbiology*, *13*. https://doi.org/10.3389/fmicb.2022.1053169]

Line 61 please update to current references

[Thank you for your comment, a 2023 paper was added:

Marchuk, S., Tait, S., Sinha, P., Harris, P., Antille, D. L., & McCabe, B. K. (2023). Biosolids-derived fertilisers: A review of challenges and opportunities. *Science of The Total Environment*, *875*, 162555. https://doi.org/10.1016/j.scitotenv.2023.162555]

Line 68 please update references

[Thank you for your comment, a 2024 paper was added:

Elgarahy, A. M., Eloffy, M. G., Priya, A. K., Yogeshwaran, V., Yang, Z., Elwakeel, K. Z., & Lopez-Maldonado, E. A. (2024). Biosolids management and utilizations: A review. *Journal of Cleaner Production*, *451*, 141974. https://doi.org/10.1016/j.jclepro.2024.141974]

Line 76 What type of wastewater treatment? Are authors referring to municipal wastewater, a specific type of industrial wastewater, etc.? The source will impact the types of organics that are present. Need to specify the 'bioremediation approach.' Do the authors mean addition of organic content to enhance indigenous microbial activity (biostimulation)n or to enhance added (i.e., foreign) microbes?

[Thank you for this comment. The organic waste is referring to class A biosolids from a municipal wastewater treatment plant. The biosolids was used for biostimulation. DC water class A biosolids, which goes through a thermal hydrolysis processes and digestion to meet the stringent requirements to be commercially sold as soil amendment. Furthermore, the municipality also has stringent requirements for the inflow industrial wastewater thus making sure that the biosolids are below the acceptable levels. This information has been added in the text now read as:

Lines 76-78: The overall goal of this study was to assess the applicability of class A biosolids originating from municipal wastewater treatment as a sustainable bioremediation approach for a TCE-contaminated groundwater site, by biostimulation.

Lines 112-118: Biosolids were acquired from Blue Plains Advanced Wastewater Treatment plant, Washington, D.C., and the toxicity characteristics leachate procedure (TCLP, SW-846 test method 1311, US EPA, 2015) was conducted, in a commercial laboratory, to ensure the safety of the waste product to be used based on the Resource Conservation and Recovery Act (RCRA) levels. Biosolids used in this study, go through thermal hydrolysis and digesters to meet the most stringent requirements and is available commercially as Bloom soil amendment and are pathogen free (Barber, 2017; Wang et al., 2018; Bloomsoil.com, 2022)]

Line 78 when was the biowall installed? What did it contain?

[Thank you for this comment. The details of installation of the PRB has been previously published elsewhere (Saffari Ghandehari et al., 2023). From Saffari Ghandehari et al., 2023:

The biowall was designed to be a channel with the dimensions of 100.5 m (length) by 0.7 m (width) by 5.5–7 m (depth), enclosing groundwater flow from the main contaminant source (Figure [2a](https://onlinelibrary.wiley.com/doi/full/10.1002/rem.21764#rem21764-fig-0002)). Bench-scale studies were conducted and calculated the average coefficient of loss for the proposed filling material to be 0.25, which was accounted for when calculating how much material would be needed for the biowall installations (BMT Designers & Planners Inc., [2017b](https://onlinelibrary.wiley.com/doi/full/10.1002/rem.21764#rem21764-bib-0004)). Wood mulch from a variety of trees and vegetation in the BDRLF was prepared at the BARC compost research facility (CRF). Approximately 765 m^3^ of the compost with municipal leaf content and food residuals (90%–10%, respectively) was screened and moved to the CRF (BMT Designers & Planners Inc., [2018](https://onlinelibrary.wiley.com/doi/full/10.1002/rem.21764#rem21764-bib-0007); Niño de Guzman, [2018](https://onlinelibrary.wiley.com/doi/full/10.1002/rem.21764#rem21764-bib-0042)). Approximately 1660 tons of washed concrete sand were used as a part of the filling material for the biowall. A sample of the selected filling materials was tested for hazardous waste characteristics using the toxicity characteristics leachate procedure (TCLP) and found to be nonhazardous (BMT Designers & Planners Inc., [2017b](https://onlinelibrary.wiley.com/doi/full/10.1002/rem.21764#rem21764-bib-0004)).

A track-mounted single-pass trencher was used to excavate 5.5 m below ground surface (bgs) for the biowall installation. It was ensured that the biowall was set at least 15 cm into the Arundel Clay formation (Figure [2b](https://onlinelibrary.wiley.com/doi/full/10.1002/rem.21764#rem21764-fig-0002)) by inspecting the cutting materials. A Komatsu 350 excavator was used to add the material into the biowall. The excavated soil was moved upgradient of the biowall by the conveyor belt mounted on the trencher unit and remained in the biowall clearing area. The biowall fill material was compacted by the excavator and 0.3–0.46 m of the native soil was added to the top of the biowall trench. Volatile organic compounds (VOCs) were not detected in soil samples excavated from the trench, so these were used for grading the biowall clearing area (BMT Designers & Planners Inc., [2017b](https://onlinelibrary.wiley.com/doi/full/10.1002/rem.21764#rem21764-bib-0004)).

This information was summarized and included in section 2.1. Site description.]

Line 81 if activity was from indigenous community, why did authors think there would be TCE degraders?

[Thank you for this comment. This was tested in 2016 and it was shown that there are indigenous TCE degrading bacteria present in the site and this data has been previously published (Saffari Ghandehari et al., 2023) and is now addressed in the text that now reads as: Microbial communities from the site were characterized and the impact of the mixed trench filling material on the indigenous microbial community of groundwater, including the dechlorinating bacteria (this was shown in Saffari Ghandehari et al., 2023) were assessed.

Saffari Ghandehari, S., Cheng, S.-H., Hapeman, C. J., Torrents, A., & Kjellerup, B. V. (2023). Ensuring the continued success of a mulch biowall at a trichloroethylene-contaminated superfund site: Lessons learned. *Remediation Journal*, *33*(4), 323–337. https://doi.org/10.1002/rem.21764 ]

Line 84: Upcycled? Not sure what that is meant by that. Wastewater has been studied as amendments before, just not injected into the ground. If this was municipal wastewater, did authors purify to make sure no pathogenic organisms were being added? If wastewater was not sterilized beforehand, did authors track impact of added organisms on indigenous culture and remediation (wastewater, even treated wastewater, contains significant activity).

[As explained in the previous comments, the Biosolids used in this study are class A biosolids, originated in an advanced wastewater treatment plant with the thermal hydrolysis and anaerobic digestion process and complies with requirements for field application in MD. This means that the pathogens were killed through the process via pasteurization.

This has been addressed in the text:

Lines 112-118: Biosolids were acquired from Blue Plains Advanced Wastewater Treatment plant, Washington, D.C., and the toxicity characteristics leachate procedure (TCLP, SW-846 test method 1311, US EPA, 2015) was conducted, in a commercial laboratory, to ensure the safety of the waste product to be used based on the Resource Conservation and Recovery Act (RCRA) levels. Biosolids used in this study, go through thermal hydrolysis and digesters to meet the most stringent requirements and is available commercially as Bloom soil amendment and are pathogen free (Barber, 2017; Wang et al., 2018; Bloomsoil.com, 2022)]

Line 89: If saying one of the 100's of contaminated sites, need updated references. Citation from 2011 is no longer applicable as sites may have been remediated.

[Thank you for your comment. ATSDR reference is from 2019, the 2011 reference is the USDA report regarding this very site, which explains the condition of the site before remediation efforts.]

Line 94 What was the active ingredient in the PRB? It will speak to the aquifer characteristics. Has the reactive material been replaced since 1994?

[Thank you for your comment this has been updated in the text and now reads as follows: “In 2013, a permeable reactive barrier (PRB), also known as a biowall, was installed with compost, sand, and mulch, between the plume and the creek to promote the activity of dehalogenating bacteria and enhance TCE dehalogenation in the site (Niño de Guzmán, Hapeman, Millner, McConnell, et al., 2018; Saffari Ghandehari et al., 2023).” The biowall was installed in 2013 and the materials have not been replaced since then.]

Line 98 Be careful of phrasing. If TCE is target, the presence of VC would be a degradation intermediate. Cannot automatically say 'incomplete TCE' dehalogenation as TCE is not present as TCE. Another issue is that VC is typically considered a 'dead end' metabolite as it is very stable and resistant to most organisms. Furthermore, depending on the thickness of the biowall, VC downgradient could be a result of migration away from the active community in the biowall.

[Thank you for your comment. This has been addressed in the text and now reads as: The monitoring data from 2013 to 2019, showed that while there is a significant decrease in the TCE concentration, the VC concentration downgradient from the PRB had increased, indicating incomplete dechlorination of chlorinated solvents by the microbial community in the PRB.]

Line 104 is the 2020 trench the focus of this study? If not, cannot say statement in last introduction paragraph about being the 'first study'. If yes, need to clearly state that 2020 trench is the focus of the manuscript.

[Thank you for your comment. Yes, the 2020 trench is the focus of this study. This has been addressed in the text and now reads: “To increase the efficiency of the bioremediation process and to increase the residence time, a smaller trench was installed in 2020, approximately 9 m upgradient from the PRB, which is the focus of this study.”]

Line 113 reference form 1994 is not needed.

[Thank you for your comment, this reference was deleted]

Line 137 Who was the third party? Was it an EPA approved facility? Did the facility also do the analysis? How many replicates were used for each sample location?

[Thank you for your comment. The third-party company was a consulting company that was contracted directly by USDA. The analyses were done in a commercial lab and was contracted by the third-party consulting company.]

Line 220L If say 'significantly' have to have run the statistics and include (p<0.05)

[Thank you for your comment. This was addressed in the text and now reads: Results showed that the TCE levels had decreased notably, when groundwater encountered the trench.]

Stopped at 220 as was sufficient comments for a reject rating.

[We thank the reviewer for the comments. We have continued to revise the manuscript according to the comments incl references, more concise statements etc. after line 220]

Reviewer #2:

The manuscript describes an interesting approach to eliminate trichloroethene in groundwater in situ, the remediation system is bases in organic waste, the analyses of microbial community reveal key metabolic process implicated in trichloroethene dehalogenation, significant trichloroethene concentrations reductions were observed.

Authors´ need address following commentaries

Commentaries:

Line 23, explain why trichloroethene is an environmental polluted, which process release it to the environment

Line 24 and 25, "trichloroethene (TCE)" could be just "TCE"

[Thank you for your comment. This has been addressed in the text.]

Line 54, add a space in "VC(often"

[Thank you for your comment. This has been addressed in the text.]

Line 64, arrange references in chronological

[Thank you for your comment. This has been addressed in the text.]

Line 71, arrange references in chronological

[Thank you for your comment. This has been addressed in the text.]

Line 96, check format in reference "Niño de Guzmán, Hapeman, Millner, McConnell, et al., 2018"

[Thank you for your comment. This has been addressed in the text.]

Line 97, "TCE concertation" could be "TCE concentration"

[Thank you for your comment. This has been addressed in the text.]

Line 113, arrange references in chronological

[Thank you for your comment. This has been addressed in the text.]

Line 123, check format in reference "Niño de Guzmán, Hapeman, Millner, McConnell, et al., 2018"

[Thank you for your comment. This has been addressed in the text.]

Figure 1 caption, define acronyms

[ this has been addressed in the figure and the caption, please refer to responses to the reviewer 3 comments.]

Line 53, "spp. ("

[Thank you for your comment. This has been addressed in the text.]

Check references format in table 1, check table numbering

Line 155, ")."

[Thank you for your comment. This has been addressed in the text.]

Line 166, "run ("

[Thank you for your comment. This has been addressed in the text.]

Line 168, ")."

[Thank you for your comment. This has been addressed in the text.]

Reviewer #3:

This manuscript reports the potential of organic waste originating from wastewater treatment for a TCE-contaminated groundwater bioremediation. The organic amendment together with lime and biochar were effective in TCE dehalogenation, decreasing the TCE levels to below the detection limit. However, it is not clear whether it was achieved through a biological and/or physical process.

Information in line 154 and 166 should be completed.

[Thank you for your comment.The primer sequence, the thermal cycler program as well as the standard curve range are now mentioned in Table 1.]

Sentence of line 249-251 should be rewritten.

*“At twelve months, the concentrations of DCE and VC decreased to below detection and*

*2.7 μg/L respectively from 43 and 19 μg/L respectively), with ethene levels increasing to 23 μg/L (from below detection at nine months).”*

[Thank you for pointing this out. The sentence is confusing and has been revised. It now reads: “At twelve months, the concentrations of DCE and VC decreased to below detection and 2.7 µg/L respectively (from initial levels of 43 and 19 µg/L, respectively), while ethene levels rose to 23 µg/L (from the initial below detection limit value, at nine months).]

Figure 1, the overall design and legends must be dramatically improved. All the nomenclature used in this figure should be spread along the whole manuscript to ensure a standard name for each sampling point.

[We appreciate the comments and recommendations. The sampling locations marked at Figure 1 refer to the soil samples that were collected. These are different from the groundwater sampling locations described in Figure 3. Therefore, the sampling points from Figure 1 do not appear in Figure 3. We have clarified this in M&M as well as in Figure 1/legend as well as Figure 3/legend.

Figure 1 and legend now looks as follows:

Figure 1. Map showing the BDRLF site, location of the PRB, trench, and the monitoring wells as well as the plume and the soil sampling locations are shown. TW: transect well, BW: biowall well (modified from BMT Designers & Planners Inc., 2018).

Figure 3 legend now looks as follows:

Figure 3. A) Concentration of chlorinated solvents, measured in groundwater samples, inside wells, from upgradient the trench to downgradient the PRB, 9 months after installation of the trench (September 2020 monitoring event). B) Concentration of the chlorinated solvents over time, in one monitoring well (TW5) between the trench and the PRB (in groundwater samples). The sampling names for soil and groundwater, respectively, have been clarified throughout the manuscript as recommended by the reviewer].

Figure 3, following the map depicted in Figure 1, the X-axis names are not properly placed. This fact prevents the reader from following the whole paper in a smooth and rational manner. The point designed as "MW6" should be "TW6" according to Figure 1. Moreover, according to Figure 1, the "Trench" point represented in Figure 3 should be "TW5"; the "TW5" point in Figure 3 should be "TW4", according to Figure 1. Authors must correct these errors and double check along the text for the proper use of the standard nomenclature of each sampling point.

[We appreciate the comments and recommendations. Figure 1 was revised according to the comments above. It now contains the sampling location number/names. These same names have been used in Figure 3 and in the remaining parts of the manuscript, so it is easier to understand for the reader.]

Figure 4, the graphical legend of the figure is extremely confusing for the reader, including the geometrical symbols and the color codes used. Please, limit the legend to specific symbols for each sampling point and deep, and use color strands to represent every group (DO NOT USE the same symbol to depict the color codes). When representing the sampling points, please USE the same nomenclature as before, referring the sampling locations as TW followed by numbers and avoiding the use of "Down trench" or "Up trench".

[Thank you for your comment. An explanation was added in the legend of Figure 4 to make it easier to navigate. The caption of Figure 4 now reads: Figure 4. The similarity of microbial communities in soil samples from the site. Determined by PCoA analysis based on unweighted UniFrac dissimilarity matrix. Different color shades are referring to each sampling location, whereas the different shapes are referring to the depths at which the sample was taken.]

Also in Figure 4, the authors claimed that "microbial analysis of the soil samples collected from the site showed that the organic waste amendment significantly affected the Indigenous microbial community", and in my opinion this is not so clear. For example, PRB data is completely different from others, and the PCA analysis also showed that TW4 data is closer to UP trench than to Down trench. Is this a factor related to the intrinsic lack of specificity of the rRNA sequencing technique to characterize microbial populations?

[Thank you for pointing this out. We apologize for the lack of clarity in the text. All samples were sequenced together and underwent the same QA-QC approach thus there should not be an issue with the approach or sequencing technique. We have removed this sentence.]

Figure 5, legend description should be improved. What is "(A)" and "(B)"?

[Thank you for your comment. We have deleted “A” and “B” in the legend.]

Results and Discussion section, genes should be in italic.

[Thank you for your comment. This has been corrected throughout the manuscript.]

Line 399-401, sentence "Increased numbers of obligate dehalogenating bacteria and the increase in TCE dehalogenation downgradient from the trench suggests a positive impact from the organic waste amendment on TCE dehalogenation at this site." This sentence is supported by Figure 5, but Figure 6 showed that the relative abundance of dehalogenase enzymes decreased. How could authors explain this fact? Could it be through TCE sorption to the biochar? Schreiter et al. reported that biochar show stronger sorption for TCE (about 50% based on solubility-normalized Freundlich coefficients) ([doi.org/10.1016/j.chemosphere.2018.03.173](http://doi.org/10.1016/j.chemosphere.2018.03.173))

[Thank you for bringing this up. The way that this was written was unclear.

“Could it be through TCE sorption to the biochar?”

The activity results (Figure 3) showed that not all of the TCE was adsorbed to biochar in the trench, since degradation products formed Down Trench. The presence of TCE biodegradation products indicates that dehalogenation was happening inside the trench and downgradient from the trench.

In this situation, we compare to Up-trench as a control, since this location is not impacted by the trench+filling material thus we compare Trench to Up-trench and we compare down-trench to up-trench.

In one case 3.8.1.5 decreased in Trench and Down Trench samples

Figure 5 shows the absolute numbers of dehalogenating bacteria whereas, Figure 6 shows the relative abundance of the enzymes. The statement from the reviewer seems to be referring to a comparison of the Up-trench to the trench, since Up-trench has a lower relative abundance of two dehalogenase enzyme, and higher abundance of one dehalogenase enzymes compared to Trench (this can be seen by the lighter colors).

Reviewer #5:

The article Use of upcycled organic waste for bioremediation of groundwater contaminated with chlorinated solvents presents a very complete study on the utilization of upcycled organic waste for bioremediation of TCE. TCE is the parent compound in the study so to speak and the authors also studied the fate of the DCE and VC that resulted from the biodegradation of TCE. It was also mentioned that ethene was also present, thus suggesting the complete biodegradation of TCE. Modern tools for the detection of genes and microorganisms were used in the study and supported the conclusions of the authors on the biodegradation of the chlorinated compounds as a result of the development of a microbial community with dehalogenating bacteria. The work explains clearly the origin of the organic waste used and addressed the concerns on the possible negative effect of toxic compounds present in the organic waste used to promote the biodegradation of the TCE. The role of dehalogenating bacteria and possible interactions with methanogens was also discussed as well as the importance of maintaining under vigilance the presence of cobalamins to support the process.

Since my point of view this work offers more than sufficient information on the approach for bioremediation of groundwater polluted with these organic compounds. It is indeed (as mentioned by the authors) a low-cost approach for bioremediation and the work presented shows that taking care of the origin and treatment of the organic waste used these toxic compounds can be readily biodegraded and valuable microbial communities can be promoted.

Unless the editors consider that the document needs editing, I do not have any observation other than a light improvement on Figure 4 and Figure 6. For example, I am not sure if some symbols on figure 4 need further explanation and Figure 6 is not completely clear, particularly in the rows.

[Thank you for the supportive comments. Based on the other review comments text and figure legends have been revised thus also addressing the comments brought up here.]
